# Lack of adherence to hypertension treatment guidelines among GPs in southern Sweden-A case report-based survey

**DOI:** 10.1186/1471-2296-13-34

**Published:** 2012-07-09

**Authors:** Rickard Ekesbo, Patrik Midlöv, Sofia Gerward, Kristin Persson, Christina Nerbrand, Lennart Johansson

**Affiliations:** 1 General Practice/Family Medicine, Department of Clinical Sciences, Faculty of Medicine, Lund University, Malmö, Sweden; 2 The R&D Department of Primary Care, Malmö, Region Skåne, Sweden; 3 Cardiovascular Epidemiology, Department of Clinical Sciences, Faculty of Medicine, Lund University, Malmö, Sweden; 4 Dalby Primary Health Care Centre, Skolgatan 1, S-240 10, Dalby, Sweden

**Keywords:** Hypertension, Adherence, Guidelines, Treatment, Primary care

## Abstract

**Background:**

General practitioners (GPs) often fail to correctly adhere to guidelines for the treatment of hypertension. The reasons for this are unclear, but could be related to lack of knowledge in assessing individual patients' cardiovascular disease risk. Our aim was to investigate how GPs in southern Sweden adhere to clinical guidelines for the treatment of hypertension when major cardiovascular risk factors are taken into consideration.

**Method:**

A questionnaire with five genuine cases of hypertension with different cardiovascular risk profiles was sent to a random sample of GPs in southern Sweden (*n* = 109) in order to investigate the attitude towards blood pressure (BP) treatment when major cardiovascular risk factors were present.

**Results:**

In general, GPs who responded tended to focus on the absolute target BP rather than assessing the entire cardiovascular risk factor profile. Thus, cases with the highest risk of cardiovascular disease were not treated accordingly. However, there was also a tendency to overtreat the lowest risk individuals. Furthermore, the BP levels for initiating pharmacological treatment varied widely (systolic BP 140-210 mmHg). ACE inhibitors (70%) were the most common first choice of pharmacological treatment.

**Conclusion:**

In this study, GPs in Southern Sweden were suggesting, for different cases, either under- or overtreatment in relation to current guidelines for treatment of hypertension. On reason may be that they failed to correctly assess individual cardiovascular risk factor profiles.

## Background

High blood pressure (BP) is an important risk factor for cardiovascular disease [1]. In accordance with official guidelines [2], the overall cardiovascular risk should be taken into account before pharmacological treatment of high BP begins. There are a number of risk factors for developing manifest cardiovascular disease, most important of which are age, family history, gender, smoking, diabetes, hypercholesterolemia, low physical activity and low consumption of fruit and vegetables [3].

In spite of clear guidelines on the treatment of high BP and the availability of a number of tools for risk assessment, there is large variation in the way that different GPs initiate treatment for high BP. Furthermore, a large proportion of patients with high BP receive no or inadequate treatment [4].

In a previous study from our group, only 20% of patients treated from hypertension in primary care in southern Sweden reached the currently recommended target levels (<140/90 mmHg) [5]. This result illustrated the need for continued follow-up of defined groups of patients in order to improve the quality of care.

Furthermore, three main barriers towards implementing guidelines were identified among GPs: doubt concerning patient motivation, patient age and absence of other risk factors [6].

These previous findings reflect the problem that while guidelines are clear and concise, real patients are, in their nature, much more complex. The aim of this study was to investigate how GPs in southern Sweden adhere to clinical guidelines for the treatment of hypertension, using a case-based questionnaire to reflect patients' complex nature and taking major cardiovascular risk factors into account.

## Methods

### Participants

Skåne County is situated in the southernmost part of Sweden and has approximately 1,150,000 inhabitants. Primary care is provided by approximately 600 GPs, of whom 420 are employed by public primary health care centres. Of these 90 primary care centres, 24 centres (employing a total of 109 GPs) were randomly selected for participation in the survey and is thus believed to be a cross-section of the GP's of the area. An invitation letter, including self-administered questionnaires (see below) was sent to the head of each primary health care centre during 2006. Non-responders received telephone reminders. For each primary health care centre agreeing to participate in the survey, all physicians working at the centre were asked to fill out questionnaires on attitudes towards current guidelines [1,2] and general practice in the treatment of high BP.

No data were collected for the 19 GPs who did not fill in the questionnaires.

### Questionnaires

The survey included two postal questionnaires. Sex and information on professional experience was recorded for all GPs. The results of the first questionnaire have previously been published [6]. The second questionnaire included four questions based on five genuine cases of high BP with different cardiovascular risk profiles (see below). The five cases were based on true cases and intended to represent reasonable variation of hypertensive patients with different risk profiles common in primary care practice. Thus, the cases represent various levels of risk rather than being absolutely representative for the hypertensive population of the region. Such a cohort could possibly present a different pattern but might also give a less clear picture for decision-making regarding hypertension.

The following four specific questions were used for each case:

1. Do you find that drug treatment is indicated for this patient?

2. If so, what is your first choice of drug class for treatment?

3. What is your target BP?

4. What other cardiovascular disease (CVD) risk factors do your think should be considered when choosing an intervention?

Possible answers to the first question were “yes” or “no”. The second question included six alternative answers: “beta blockers”, “diuretics”, “ACE inhibitors”, “angiotensin receptor blockers (ARBs)”, “calcium channel blockers” and “other”. Questions 3 and 4 were open, without predefined answers to choose from; however reported CVD risk factors (question 4) were later categorized into four groups: hyperglycemia or diabetes, smoking, hyperlipidemia and obesity.

Data for each case were registered separately. The cases were assessed using two different risk assessment instruments: the SCORE, which is intended to assess cardiovascular disease mortality risk in patients without manifest cardiovascular disease and which is based on subject gender, age, systolic blood pressure, smoking status and cholesterol levels [2]; and the joint European Society of Hypertension (ESH) and European Society of Cardiology (ESC) risk assessment instruments, which take into account other risk factors and which also cover patients with manifest cardiovascular complications [1].

### Statistical methods

Only descriptive data were used in this study.

## Results

The results are presented as individual cases, followed by interpretation of the results and adherence to guidelines according to the SCORE and ESC scoring systems. A short version is shown in Table 1.

**Table 1:** The cases in short form with risk factors and response rate for treatment

**Case**	**SBP/DBP**	**Diabetes**	**Total Cholesterol**	**Target organ damage**	**Heredity**	**Score**	**ESC**	**Proportion treating (%)**
1	160/90	0	5.6	0	0	0-1	1	92
2	160/95	0	6.7	LVH	0	2	2	99
3	150/80	+	5.0	0	0	NA	2	94
4	150/80	0	4.0	CABG	+	NA	3	70
5	155/85	0	6.2	?	?	NA	2	58

LVH = Left ventricular hypertrophy. CABG = Coronary artery bypass graft.

### Case 1 facts: female, age 45 years, BP 160/95

Family history of cardiovascular disease. Non-smoker. Married with two children. Administrative work with low stress. Exercises three days a week. Previously had high blood pressure about 10 years ago, as measured by her occupational health physician and received drug treatment for a short period. BMI: 25 kg/m^2^. ECG: normal. Routine tests (full blood count and routine biochemistry): no abnormalities detected (NAD). Serum cholesterol: 5.6 mmol/l.

### Case 1 risk assessment

Case 1 represents a case of low CVD risk, being a non-smoking female with mild hypertension. According to the risk assessment, case no 1 has a SCORE of 0-1 and an ESC score of 1. Thus, the risk for manifest CVD disease within 10 years is very low (1-2%) and blood pressure treatment may not be indicated.

### Case 1 results

In this case, 92% of the GPs found a drug treatment indication for hypertension. The target systolic blood pressure was 140 for 83% of responders and the diastolic blood pressure 90 in 46% of responders.

Among those who found a drug treatment indication, 73% selected diuretics as their first choice of treatment and 25% ACE inhibitors or ARBs. 28 and 29% of GPs, respectively, thought that the cholesterol levels and the weight also should be treated.

#### Case 2 facts: female, age 54 years, BP 160/95

No family history of cardiovascular disease. Non-smoker. Married with two children. Originally from Greece. Manual worker. Slight obesity. No daily exercise. Blood pressure repeatedly measured to be 160/95. Patient reports occasional palpitations and decreasing physical condition. Physical examination reveals a murmur in the left carotid artery. BMI: 30 kg/m^2^. ECG: suspected left ventricular hypertrophy (LVH). Routine tests: NAD. Serum-cholesterol: 6.7 mmol/l.

### Case 2 risk assessment

This patient represents a medium cardiovascular risk, having suspected left ventricular hypertrophy. She has a SCORE of 2 and an ESC score of 2. With hypertension in the medium range, this patient should be treated. Target organ damage (TOD) should be addressed.

### Case 2 results

Almost all GPs (99%) chose to treat the blood pressure. Of these, 43% selected ACE-I as their first choice of treatment, followed by diuretics (23%) and beta blockers (10%). 41% of the GPs thought that the raised serum cholesterol should be treated.

#### Case 3 facts: male, age 56 years, BP 150/80

Family history of diabetes, but not heart disease. Non-smoker. Married with two children. Taxi driver. Low exercise levels. Diabetes since the age of 5; treated with diet and oral antidiabetics; no organ complications. Blood pressure measured to be 150/80 (by his GP) and 145/80 (by his diabetes nurse). BMI: 28 kg/m^2^. ECG: normal. Routine tests: NAD. Serum creatinine: 74 mmol/l. Microalbuminuria: negative. Serum cholesterol: 5,0 mmol/l. HgbA1c: 6.8%.

### Case 3 risk assessment

This case represents a medium cardiovascular risk with diabetes and an unsatisfactory blood pressure. Due to the presence of diabetes mellitus type 2 SCORE cannot be assessed. The ESC score is 2.

### Case 3 results

94% of the GPs wanted to treat the hypertension, the majority of whom (79%) selected ACE inhibitors as the first choice of treatment. The target BP levels for most was 130/80, which is the recommended value for diabetic patients [1]. 26 and 22% of the GPs, respectively, wanted to increase the treatment of hyperglycemia and hyperlipidemia, whereas 41% wished to treat the patient's overweight.

#### Case 4 facts: female, age 62 years, BP 150/80

Family history of coronary heart disease on the maternal and paternal sides. Married. Newspaper delivery person. Coronary artery bypass graft (CABG) five years ago due to angina problems. Current medication: metoprolol 50 mg × 1, pravastatin 40 mg × 1, felodipin 10 mg × 1 and aspirin 75 mg × 1. Smokes 20 cigarettes per day. No current angina. Home-measured BP around 140/80; nurse-measured blood pressure 150/80; GP-measured BP 145/80. BMI: 25 kg/m^2^. ECG: normal. Routine tests: NAD. Serum creatinine: 67. Microalbuminuria: negative. Serum cholesterol: 4.0 mmol/l.

### Case 4 risk assessment

This is a case of high cardiovascular risk due to manifest coronary artery disease, continued smoking, and elevated blood pressure. SCORE cannot be assessed as manifest coronary disease is present. The ESC score is 3.

### Case 4 results

70% of GPs wanted to increase hypertension treatment, with ACE inhibitors being the most common choice (55%), followed by thiazides (28%). Smoking was identified as a treatable risk factor by 85% of GPs.

#### Case 5 facts: male, age 77 years, BP 170/95

Retired, married, physically active man. No family history of cardiovascular disease. Non-smoker; drinks alcohol very modestly. Exercises regularly. Healthy diet. No other diseases. Presently has occasional discomfort in his left arm; in connection with this, his BP is measured and found to be high. Exercise stress ECG: normal. Blood pressure, as measured by a nurse, is 170/95 and 150/85; at the next visit, his BP is 155/80. BMI: 22 kg/m^2^. ECG: normal. Routine tests: NAD. Serum cholesterol: 6.2 mmol/l.

### Case 5 risk assessment

This is a case of medium cardiovascular risk with a medium hypertension levels. SCORE cannot be assessed in patients of this age. The ESC score is 2.

### Case 5 results

58% of GPs wanted to start treatment with the aim of achieving the target BP (mean 140/86). Of these GPs, 58% selected thiazides as their first choice of treatment; 21% chose ACE inhibitors and 17% beta blockers 17%. 18% of GPs wanted to treat the raised cholesterol levels.

#### Summary of results

Tendency to under-treat was particularly evident for cases 4 and 5, with just 70 and 58% of GPs willing to treat these patients, respectively. Conversely, the present guidelines give no support for treatment in case no 1 with mild hypertension whereas the clinicians overwhelmingly chose to treat.

ACE inhibitors and thiazides were the most popular drug choices, in accordance with guidelines and local recommendations. For cases 1 and 5 (a young woman and an old man, respectively), thiazides were the preferred drugs. For case 2, who had a target organ damage (LVH), a rather low proportion of GPs (43%) were choosing ACE inhibitors, which have best evidence for effectiveness for this condition.

There were a number of suggestions for treating other risk factors, with concentration on smoking cessation and non-pharmacological treatment of obesity. In case 3, a patient with type 2 diabetes, the responses show that the guidelines concerning treatment of hypertension when diabetes is present were well understood by GPs.

## Discussion

The results of the present study, showed under- as well as overtreatment in the majority of the presented cases, emphasizing the need for better use of guidelines for the treatment of hypertension.

Case-based questionnaires aim to illustrate how judgment concerning treatment is made in cases of different severity. The cases provide a description of a real clinical challenge in a realistic clinical setting. It is important to receive a high response rate, and in order to achieve this the amount of information for the case presentation has to be reasonable. In this study, the participating GPs were randomly selected. Together with the high participation rate, this adds to the credibility of the results.

In addition, the cases were reasonably varied in terms of overall cardiovascular risk factor burden.

Our study has some limitations. A questionnaire is not the same as clinical practice, where decisions on treatment take place in concordance with the wishes of the patient, and in itself, the questionnaire may induce a more active approach than real life. Also, although guidelines do exist, there are no correct answers as such for individual cases. It should also be pointed out that the responders completed the questionnaires during office hours with a normal patient load and may thus not have had adequate time to consider the clinical implications for each case. This may have caused inconsistencies regarding the answers.

Perceived lack of time may also show the difficulty of implementing the SCORE system as a method for use in clinical practice. It could be used as a guideline, but not for automatically translating data from population studies to clinical practice. On the other hand, cases may be used as an educational method to increase the awareness of treatment options, as well as a method of priority [7].

Assessment by the ESH and SCORE methods has been questioned, particularly their failure to indicate the same CV risk levels [8]. For example, the SCORE system does not include diabetes as a risk factor. Also, further risk assessment tools tailored to primary care have recently been proposed [9]. Regarding the generalisability of the results, the answers came from a particular setting, but the cases used should be familiar in the Western world. The results show that correctly assessing risk is difficult, affecting the consequent choice of treatment.

Finally, our questionnaire has not been validated, which makes the reproducibility of the responses uncertain. It would be difficult to find a method of validation since all information of the patient cannot be provided.

Recently, the awareness-to-adherence model has been presented [10] in an attempt to explain failures to reach the blood pressure target. This model could also show that guidelines are on their own insufficient, and should be complemented by educational efforts.

Self-report studies have described treatment in clinical practice [11,12]. However, these studies have been limited to BP targets and the reasons for not intensifying treatment. Further questions, such as drug choices and familiarity with research methods, were addressed in a large survey [13]. Some geographic differences in attitudes concerning age and the cost of medication have also been reported [14]. When co-morbidity is taken into consideration, younger physicians are more likely to follow guidelines [15], a finding we could not confirm. This may also be true for non-pharmacological recommendations [16].

Although frequently commented by our responders, we have not quantified this particularly.

A case-based method could be of help in the assessment of judgments made by the clinicians. Indeed, this has been used for other diagnoses [17], but the use of the method is rare for the treatment of high BP, and thus we find our study adding to present knowledge.

The difficulty in showing adherence to guidelines due to different risk levels was described by Milchak et al [18]. Previous studies [19] have also shown limited correlation between increased number of risk factors and increased levels of treatment. Another study [20] showed that physicians tend to focus rather on diastolic blood pressure when choosing treatment, leaving systolic hypertension less well controlled. Our findings rather contradict this and thus, our findings confirm that there is much room for improvement in the assessment of CVD risk. E.g., this is illustrated in case 5, where the low treatment rate may be due to reluctance to treat older patients, although this may be the group which would have the greatest benefit from treatment [21]. However, when it comes to treatment of the oldest, a Swedish study showed that lower systolic blood pressure may be associated with greater mortality in patients aged 85 or more. [22]

Factors causing reluctance to treat high BP may thus include hesitance about the guidelines, partly due to doubts concerning the effectiveness of additive treatment (referred to by some as “over-treatment”), difficulty in being up-to date, but also lack of time with patients and GPs concerns about lack of compliance.

The importance for adequate prevention for patients at risk is evident. This leads to the objective to increase adherence to guidelines, to put into practice what is considered an improved treatment, and in this process the cases in the questionnaire may be complemented by a structured study program showing the relative impact of different risk factors. Then it is of major importance that the guidelines give similar recommendations for treatment.

## Conclusions

The present study confirmed the lack of adherence to present hypertension guidelines. Indeed, where additional risk factors are present, the guidelines do not seem to increase the tendency to initiate treatment. It may be easier to recognise an isolated BP value than to deal with more complex patient cases in which different risk factors should be taken into consideration.

For the future, since hypertension is common in primary care, we believe research emphasis should be put on efforts to increase the awareness and thus the adherence to guidelines in this setting. Computer-based tools allowing new – medical and educational- data to be readily available could prove useful for this purpose.

## Consent

The study was approved by the Ethics committee of Lund University.

## Key points

Despite international and national clinical guidelines on the treatment of hypertension, general practitioners often fail to correctly assess the cardiovascular risk for patients in a clinical setting.

● Most GPs use target blood pressure levels but do not consider other cardiovascular risk factors.

● Both under- and overtreatment of high and low cardiovascular risk groups were seen in this study.

## Competing interest

There are no financial or other conflicts of interest.

## Authors' contributions

All authors have been active within the framework for cardiovascular prevention in Region Skåne. RE has been the main manuscript writer with co-work and input from PM. SG, KP and CN have participated in the discussions over cardiovascular prevention, the individual cases and have provided comments on the manuscript. LJ have been the provider of the cases and evaluated them in reference to guidelines and has also participated actively in producing the text in the manuscript. All authors read and approved the final manuscript.

## Pre-publication history

The pre-publication history for this paper can be accessed here:

http://www.biomedcentral.com/1471-2296/13/34/prepub

## References

[B1] EuropeanSociety of Hypertension-EuropeanSociety of CardiologyGuidelines Committee General cardiovascular risk profile for use in primary care: the Framingham Heart Study Circulation 2008 117 743 753 10.1161/CIRCULATIONAHA.107.699579 18212285

[B2] Conroy RM Pyörälä K Fitzgerald AP Sans S Menotti A De Backer G De Bacquer D Ducimetière P Jousilahti P Keil U Njølstad I Oganov RG Thomsen T Tunstall-Pedoe H Tverdal A Wedel H Whincup P Wilhelmsen L Graham IM SCORE project group. Estimation of ten-year risk of fatal cardiovascular disease in Europe: the SCORE project Eur Heart J 2003 24 987 1003 10.1016/S0195-668X(03)00114-3 12788299

[B3] Yusuf S Hawken S Ounpuu S Dans T Avezum A Lanas F McQueen M Budaj A Pais P Varigos J Lisheng L INTERHEART Study Investigators. Effect of potentially modifiable risk factors associated with myocardial infarction in 52 countries (the INTERHEART study): case-control study Lancet 2004 364 937 952 10.1016/S0140-6736(04)17018-9 15364185

[B4] Moderately elevated blood pressure 2006 2006 The Swedish Council on Technology Assessment in Health Care, Stockholm

[B5] Hedblad B Nerbrand C Ekesbo R Johansson L Midlöv P Brunkstedt I Gyllerup S Sträng B Persson R Janzon L High blood pressure despite treatment: a cross-sectional primary healthcare-based study in southern Sweden Scand J Prim Health Care 2006 24 224 230 10.1080/02813430601002134 17118862

[B6] Midlöv P Ekesbo R Johansson L Gerward S Persson KO Nerbrand C Hedblad B Barriers to adherence to hypertension guidelines among GPs in southern Sweden: a survey Scand J Prim Health Care 2008 26 3 154 159 10.1080/02813430802202111 18609250 PMC3409603

[B7] Herbert CP Wright JM Maclure M Wakefield J Dormuth C Brett-MacLean P Legare J Premi J Better Prescribing Project: a randomised controlled trial of the impact of case-based educational modules and personal prescribing feedback on prescribing for hypertension in primary care Fam Pract 2004 21 575 581 10.1093/fampra/cmh515 15367481

[B8] Lengelé JP Vinck WJ De Plaen JF Persu A Cardiovascular risk assessment in hypertensive patients: major discrepancy according to ESH and SCORE strategies J Hypertens 2007 25 757 762 10.1097/HJH.0b013e328017f6fa 17351366

[B9] D'Agostino RB Vasan RS Pencina MJ Wolf PA Cobain M Massaro JM Kannel WB General cardiovascular risk profile for use in primary care: the Framingham Heart Study Circulation 2008 117 743 753 10.1161/CIRCULATIONAHA.107.699579 18212285

[B10] Heneghan C Perera R Mant D Glasziou P Hypertension guideline recommendations in general practice: awareness, agreement, adoption, and adherence Br J Fam Pract 2007 57 948 952 10.3399/096016407782604965 PMC2084133 18252069

[B11] Frijling BD Spies TH Lobo CM Hulscher ME an Drenth BB Braspenning JC Prins SA van der Wouden JC Grol RP Blood pressure control in treated hypertensive patients: clinical performance of general practitioners Br J Gen Pract 2001 512 9 14 11271892 PMC1313918

[B12] Ferrari P Hess L Pechere-Bertschi A Muggli F Burnier M Reasons for not intensifying anti-hypertensive treatment (RIAT): a primary care anti-hypertensive intervention study J Hypertens 2004 22 1221 1229 10.1097/00004872-200406000-00024 15167458

[B13] Hyman DJ Pavlik VN Self-reported hypertension treatment practices among primary care physicians: blood pressure thresholds, drug choices, and the role of guidelines and evidence-based medicine Arch Intern Med 2000 160 2281 2286 10.1001/archinte.160.15.2281 10927724

[B14] Troein M Arneson T Råstam L Pirie PL Selander S Luepker RV Reported treatment of hypertension by family physicians in Sweden and Minnesota: a physician survey of practice habits J Intern Med 1995 238 215 221 10.1111/j.1365-2796.1995.tb00925.x 7673850

[B15] Mehta SS Wilcox CS Schulman KA Treatment of hypertension in patients with comorbidities: results from the study of hypertensive prescribing practices (SHyPP) Am J Hypertens 1999 12 333 340 10232492

[B16] Arroll B Jenkins S North D Non-pharmacological management of hypertension: results from interviews with 100 general practitioners J Hypertens 1996 14 773 777 10.1097/00004872-199606000-00014 8793701

[B17] Beaulieu MD Brophy J Jacques A Blais R Battista RN Lebeau R Physicians' attitudes to the pharmacological treatment of patients with stable angina pectoris QJM 2005 98 41 51 10.1093/qjmed/hci006 15625353

[B18] Milchak J Carter BL Ardery G Measuring adherence to practice guidelines for the management of hypertension: an evaluation of the literature Hypertension 2004 44 602 608 10.1161/01.HYP.0000144100.29945.5e 15381676 PMC1414061

[B19] Kannel WB D'Agostino RB Sullivan L Wilson PW Concept and usefulness of cardiovascular risk profiles Am Heart J 2004 148 16 26 10.1016/j.ahj.2003.10.022 15215787

[B20] Fagard RH van den Emden M Treatment and blood pressure levels in isolated hypertension and diastolic hypertension in primary care J Hum Hypertens 2003 17 680 687 10.1038/sj.jhh.1001598 14504626

[B21] Beckett NS Peters R Fletcher AE Staessen JA Liu L Dumitrascu D Stoyanovsky V Treatment of hypertension in patients 80 years of age or older N Engl J Med 2008 358 1887 1898 10.1056/NEJMoa0801369 18378519

[B22] Molander L Lövheim H Norman T Nordström P Gustafson Y Lower Systolic Blood Pressure is associated with greater mortality in people aged 85 and older J Am Geriatr Soc 2008 56 1853 1859 10.1111/j.1532-5415.2008.01948.x 18811610

